# Infection with *Mansonella perstans* Nematodes in Buruli Ulcer Patients, Ghana

**DOI:** 10.3201/eid2006.131501

**Published:** 2014-06

**Authors:** Richard O. Phillips, Michael Frimpong, Fred S. Sarfo, Birte Kretschmer, Marcus Beissner, Alexander Debrah, Yaw Ampem-Amoako, Kabiru M. Abass, William Thompson, Mabel Sarpong Duah, Justice Abotsi, Ohene Adjei, Bernhard Fleischer, Gisela Bretzel, Mark Wansbrough-Jones, Marc Jacobsen

**Affiliations:** Kwame Nkrumah University of Science and Technology, Kumasi, Ghana (R.O. Phillips, A. Debrah);; Komfo Anokye Teaching Hospital, Kumasi (R.O. Phillips, F.S. Sarfo, Y. Ampem-Amoako, O. Adjei);; Kumasi Collaborative Centre for Research, Kumasi (M. Frimpong, M. Sarpong Duah);; Bernhard Nocht Institute of Tropical Medicine, Hamburg, Germany (B. Kretschmer, B. Fleischer);; University Hospital, Ludwig-Maximilians-University of Munich, Munich, Germany (M. Beissner, G. Bretzel);; Agogo Presbyterian Hospital, Agogo, Ghana (K.M. Abass, W. Thompson, J. Abotsi);; St. George’s University of London, London, UK (M. Wansbrough-Jones);; University Children’s Hospital, Dusseldorf, Germany (M. Jacobsen)

**Keywords:** Mycobacterium ulcerans, Buruli ulcer, Mansonella perstans, co-infection, bacteria, Ghana, parasites, nematodes, filariae

## Abstract

During August 2010–December 2012, we conducted a study of patients in Ghana who had Buruli ulcer, caused by *Mycobacterium ulcerans*, and found that 23% were co-infected with *Mansonella perstans* nematodes; 13% of controls also had *M. perstans* infection. *M. perstans* co-infection should be considered in the diagnosis and treatment of Buruli ulcer.

Buruli ulcer, caused by *Mycobacterium ulcerans*, is a neglected tropical disease common in rural parts of West Africa. Infection with *M. ulcerans* causes disfiguring skin ulcers, mainly in children. The disease is highly focal, and in Ghana, cases are reported mainly from the humid and tropical southern regions, including Ashanti and Greater Accra (*1*)*.* Recent studies suggest that aquatic invertebrates serve as a reservoir for *M. ulcerans*, although complete transmission pathways remain unknown (*2*,*3*)*.* Aquatic insects infected with *M. ulcerans* can establish infection in mice by biting (*4*), but it is not clear that this is the cause of human infection (*5*)*.* In southeastern Australia, evidence has been found linking infected mosquitoes with human cases (*6*,*7*), but proof of transmission is lacking.

Residents of regions in which Buruli ulcer is endemic are frequently exposed to parasitic infections such as filariasis. In Ghana, lymphatic filariasis caused by *Wuchereria bancrofti* nematodes is found in several regions to which Buruli ulcer is endemic, such as the Upper Denkyira District in the central region of Ghana, but its prevalence is unknown (*8*). The filarial nematode *Mansonella perstans* is endemic to countries in central and western Africa; its distribution overlaps that of other filarial nematodes *W. bancrofti*, *Loa loa*, and *Onchocerca volvulus* (*9*). Infective *M. perstans* larvae are transmitted through the bite of *Culicoides* midges (Diptera: Ceratopogonidae); the larvae develop over the course of months into adult worms that reside in serous cavities, particularly in the abdomen. *M. perstans* infection is not associated with a specific set of clinical signs and symptoms, but those attributed to this infection include acute swelling in the forearms, hands, and face that recedes in a few days and often recurs; itching with or without rash; arthralgia; and eosinophilia (*9*).

During an investigation into the immunopathogenesis of Buruli ulcer, we observed *M. perstans* nematodes in preparations of peripheral blood mononuclear cells from a patient. This finding led us to consider whether this organism was involved in the transmission or pathogenesis of *M. ulcerans* disease or if the finding was incidental. We then conducted a small case–control study to investigate the frequency of *M. perstans* co-infection in patients with *M. ulcerans* disease and the effect of this co-infection, if any, on patient response to antimicrobial drug therapy.

## The Study

During August 2010–December 2012, we recruited all patients who had clinically suspected *M. ulcerans* infection and had attended a clinic in the Buruli ulcer–endemic Asante Akim North District in Ghana. Age- and sex-matched household contacts of patients were also asked to participate; all study participants were >5 years of age. The study protocol was approved by the ethics review committee of the School of Medical Sciences, Kwame Nkrumah University of Science and Technology (CHRPE/91/10).

**Figure 1:**
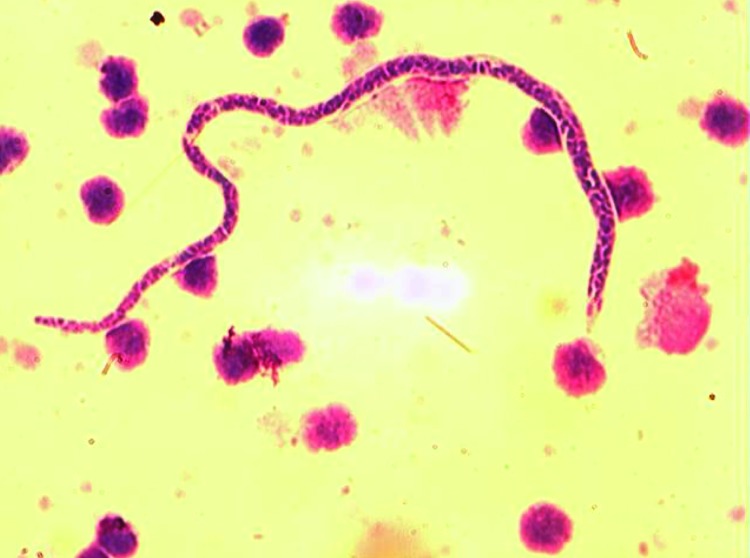
*Mansonella perstans* nematode in peripheral blood mononuclear cells from Buruli ulcer patient in Ghana. Cells were stained with Giemsa (original magnification ×1,000). *M. perstans* nematodes can be distinguished from *Loa loa* and *Wuchereria bancrofti* nematodes by relative small size, detection in blood samples obtained during the day, and lack of a sheath.

Whole blood samples were taken at baseline, at week 6, and at week 12 from 66 patients in whom the diagnosis of Buruli ulcer disease had been confirmed by PCR for the IS*2404* repeat sequence specific for *M. ulcerans* (*8*); samples were also obtained from 20 household contacts at the same intervals. The samples were heparinized, and peripheral blood mononuclear cells were separated from 10-mL samples. Filarial infection was confirmed on a blood film stained with Giemsa and Delafield hematoxylin and examined for microfilariae at ×10 and ×40 magnification (the Knott technique; *10*). *M. perstans* nematodes were distinguished from *L. loa* and *W. bancrofti* nematodes by their small size and the absence of a sheath (Figure 1). 

Patients in whom *M. ulcerans* infection was found were treated with 10 mg/kg oral rifampin and 15 mg/kg intramuscular streptomycin, administered daily at village health posts under direct observation for 8 weeks (RS8 treatment). The patients were followed up every 2 weeks in the clinic and monitored for complete healing or recurrence of skin lesions. We compared the proportion of household controls versus the proportion of Buruli ulcer patients infected with *M. perstans* nematodes and the time to complete healing of *M. ulcerans* lesions in co-infected versus monoinfected patients. Categorical variables such as sex, clinical form of *M. ulcerans* lesion, and category of *M. ulcerans* lesion were compared by using the Fisher exact test, and cumulative healing was compared by using the log-rank test.

We found all forms of *M. ulcerans* disease among the group of patients; proportions of each type and category are shown in the Table. Of 66 patients with *M. ulcerans* disease, 15 (22.7%) were co-infected with *M. perstans* nematodes, whereas 4 (13%) of 30 household controls had *M. perstans* infection (p = 0.4 by Fisher exact test). Three patients in the co-infected group and none in the *M. ulcerans*–monoinfected group reported pruritus. No other clinical signs of *M. perstans* infection were found.

**Table:** Characteristics of patients with active *Mycobacterium ulcerans* infection, monoinfected or co-infected with *Mansonella perstans*, and of household contacts, Ghana, August 2010–December 2012*

Characteristic	No. (%) persons with *M. ulcerans* infection			No. (%) household contacts, n = 30	p value
	Co-infected with *M. perstans*, n = 15	Monoinfected, n = 51	Total, n = 66		
Age, y					0.514†
<16	4 (27)	24 (47)	28 (42)	15 (50)	
16–59	11 (73)	27 (53)	38 (58)	15 (50)	
Sex					1.000†
M	9 (60)	19 (37)	30 (45)	14 (47)	
F	6 (40)	32 (63)	36 (55)	16 (53)	
Clinical form of *M. ulcerans* infection					0.049‡
Nodule	8 (53)	11 (22)	19 (29)	NA	
Plaque with edema	2 (12)	17 (33)	19 (29)	NA	
Ulcer	5 (35)	23 (45)	28 (42)	NA	
Category of *M. ulcerans* infection					0.910‡
I	9 (59)	32 (63)	41 (62)	NA	
II	4 (29)	11 (22)	15 (23)	NA	
III	2 (12)	8 (16)	10 (15)	NA	
*M. perstans* infection					0.408‡
Yes	15 (100)	NA	15 (23)	4 (13)	
No	0	NA	51 (77)	26 (87)	

*NA, not applicable. †Comparison of combined *M. ulcerans* monoinfected *and M. ulcerans* co-infected with *M. perstans* versus household contacts, determined by 2-tailed Fisher exact test. ‡Comparison of *M. ulcerans* co-infected with *M. perstans* versus *M. ulcerans* monoinfected group, determined by 2-tailed Fisher exact test.

**Figure 2:**
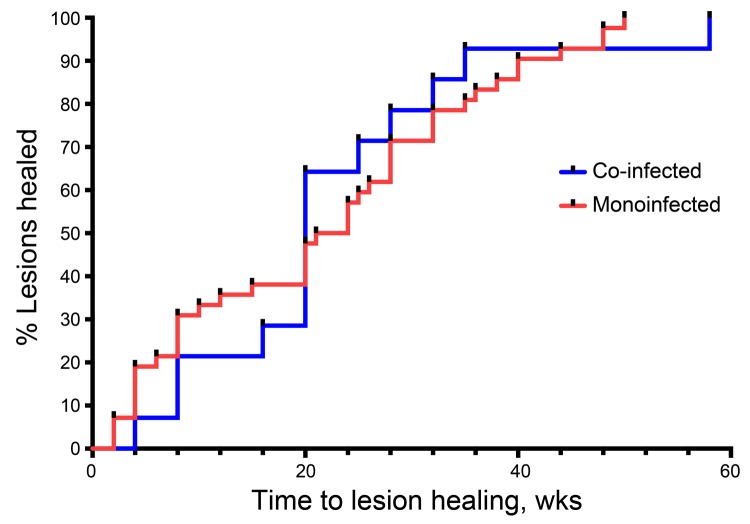
Survival analysis curve of cumulative healing for patients with *Mycobacterium ulcerans* infection who were co-infected with *Mansonella perstans* nematodes compared with those who had *M. ulcerans* monoinfection, Ghana, August 2010–December 2012. No difference in cumulative healing was found between the 2 groups (p = 0.93 by log-rank test).

All 66 patients completed RS8 treatment, but 9 were lost to follow-up during the 12-month follow-up period. Buruli ulcer lesions healed completely in 14 co-infected patients by 58 weeks (median 20 weeks, 95% CI 14.6–30.2) and in 43 monoinfected patients by 50 weeks (median 21 weeks, 95% CI 16.7–25.5). We found no difference in cumulative time to healing for co-infected versus monoinfected patients (p>0.05 by log-rank test) (Figure 2). Buruli ulcer patients who had *M. perstans* nematodes co-infection were treated with doxycycline (200 mg) and ivermectin (150 μg/kg) daily for 6 weeks, starting during the second to fourth week of RS8 treatment. Viable microfilariae were still visible in peripheral blood mononuclear cell cultures from all co-infected patients after ivermectin and doxycycline treatment, but pruritus subsided in the 3 patients who had reported it.

## Conclusions

We found co-infection with *M. perstans* in 23% of Buruli ulcer patients in a disease-endemic district in Ghana, but this prevalence was not significantly difference from prevalence among household contacts who served as controls (13%). As with Buruli ulcer, *M. perstans* filariasis is predominantly found in rural populations and infection begins in childhood; the highest infection rates are found in children 10–14 years of age (*11*), similar to those for children at highest risk for *M. ulcerans* infection. *M. perstans* infection occurs in Ghana and was seen in the Volta region of Ghana around Hohoe during the 1990s, but its prevalence is unknown (*12*), and no information is available about the average number of worms per infection. In Uganda, prevalence of *M. perstans* infection has been found to range from 0.4% to 50% (*13*). 

*M. perstans* nematodes are transmitted by the bites of *Culicoides* midges, but it is not known whether *M. perstans*–infected midges can be co-infected with *M. ulcerans*. In a guinea pig model, skin penetration was shown to be a requirement for establishment of *M. ulcerans* disease (*14*), and it has been postulated that mosquito bites cause *M. ulcerans* disease in Australia (*6*)*.* These organisms might share a common route of transmission, but our findings in this small study do not support this concept. 

Our findings suggest that *M. perstans* nematodes are common in rural Ghana and coincidentally infect patients with *M. ulcerans* disease, necessitating the consideration of these organisms in the management plan of Buruli ulcer patients. Although often asymptomatic, *M. perstans* infection may cause eosinophilia, subcutaneous swellings, aches, pains, and skin rashes in a considerable proportion of patients (*9*)*.* Because filarial nematodes are known to polarize the host immune responses from T-helper type 1 cells needed for protection against mycobacterial infections, toward humoral and T helper type-2 mediated immunity, we plan to undertake a study to investigate this interaction.
